# Children’s perspectives on being absorbed when reading fiction: A Q methodology study

**DOI:** 10.3389/fpsyg.2022.966820

**Published:** 2022-10-06

**Authors:** Anežka Kuzmičová, Markéta Supa, Martin Nekola

**Affiliations:** ^1^Institute of Czech Language and Theory of Communication, Faculty of Arts, Charles University, Prague, Czechia; ^2^Institute of Communication Studies and Journalism, Faculty of Social Sciences, Charles University, Prague, Czechia; ^3^Institute of Sociological Studies, Faculty of Social Sciences, Charles University, Prague, Czechia

**Keywords:** absorption, children, Q methodology, reading, child-centered approach, literacy, fiction, subjectivity

## Abstract

Research in the intersections of literature, media, and psychology increasingly examines the absorbing story experiences of adult readers, typically relying on quantitative self-report questionnaires. Meanwhile, little work has been done to explore how being “lost in a book” is experienced by children, despite the phenomenon’s importance for literacy education. Such work requires tools that are more inductive and child-centered than questionnaires. We have conducted a Q methodology study with participants aged 9–12 (*n* = 28), exploring how it feels for them when the mind and body are attuned to a story and how different facets of absorption (e.g., mental imagery, emotional engagement) inform the experience. Participants numerically sorted 24 cards expressing inner states and expectations relating to book-length fiction reading and were subsequently interviewed regarding their sorting choices. The cards were generated inductively based on preliminary research (focus groups, individual interviews, observations). By-person factor analysis of the sortings combined with reflective thematic analysis of the post-sorting interviews revealed four distinct reader subjectivities, or perspectives: Growth, Confirmation, Attachment and Mental Shift. Crucially, the children in these groups differed as to prominent dimensions of absorption but also as to the overall place of reading in their inner and everyday lives. Based on the four perspectives, we demonstrate that children have varied ways of being absorbed when reading fiction, and reflect on the affordances of Q methodology as a suitable child-centered approach to studying the subjective experiences of reading.

## Introduction

What does fiction reading feel like in the upper primary years, when it is still a relatively new skill? Who gets absorbed, “lost in a book” (Kuijpers et al., 2021) easily, and how can this go about? In the age of 9–12 years, learning to engage with book-length narratives on one’s own can be essential to developing a reading habit and reaping its various benefits for future life (Mar and Oatley, 2008; Locher and Pfost, 2020; Torppa et al., 2020). Existing research on the everyday subjective experiences of literacy in this period largely focuses on how reading behaviors, motivations, and reader identities are shaped by teachers, peers, and family background (Compton-Lilly, 2007, 2016; Merga, 2017; Scholes, 2018). Children’s subjective experiences of the independent reading process proper, and individual variations in this regard, receive far less systematic attention.

One exemplary facet of subjective readerly experience is sensory mental imagery. Reading skill and sensory imaging are known to be mutually correlated (Suggate and Lenhard, 2022) and in research with adult readers, imagery is a key dimension of the construct of absorption (Kuijpers et al., 2014, 2021) which captures deep engagement with fictional narratives. However, common mental imagery interventions aimed at young readers primarily rely on selected visualization techniques (De Koning and van der Schoot, 2013). As individuals vary in their propensity to conjure mental images in different sensory modalities (Isaac and Marks, 1994; Floridou et al., 2022), some children will inevitably be alienated by the instruction to visualize when reading – or to visualize in a particular way (Mackey, 2019). Increased general awareness of pre-existing differences in young readers’ inner lives can thus boost individual children’s chances of learning to become absorbed in books.

Systematic research into different children’s ways of absorbed reading, and the place of sensations within it, has therefore been called for by literacy scholars (Cremin et al., 2014; Wilhelm, 2016; Mackey, 2019). Our study begins to redress this research gap. It is grounded in the premise that for knowledge of individual differences to be practically applicable, it is not enough to conclude that children’s conduits into absorbed reading vary. Rather, patterns in this variation need to be examined that will help discern groups of children for whom different absorption facets combine in similar ways. To this end, we have carried out a Q methodology study with children aged 9–12 years, exploring what it is like for them when their mind and body are attuned to reading a story.

Q methodology is an inductive social science methodology (McKeown and Thomas, 2013) wherein participants sort authentic statements on a particular aspect of subjectivity (in our case: the experience of reading fiction) using a bell-shaped grid (scaled for agreement e.g., from –3 to +3). The sorts are then subjected to by-person (“Q”) factor analysis. Unlike traditional (“R”; Stephenson, 1936) factor analysis, this procedure exposes pre-existing groups of people sharing a distinct perspective, rather than pre-existing relationships between variables that are generalized to a larger population. The interpretation of these factors further draws on qualitative analyses of post-sorting interviews which elucidate the participants’ understanding of individual statements.

The research presented here is the first Q methodology study known to us focusing on children’s experiences of reading. Participants sorted statements that were extracted from preliminary qualitative research primarily consisting of creative focus groups and in-depth interviews on how stories “feel from within our bodies” (Kuzmičová et al., 2022). Four distinct perspectives emerged among the participants of the Q study: the *Growth, Confirmation, Attachment* and *Mental Shift* perspectives, respectively. These differ in absorption style but also beyond, e.g., in what drives one’s reading forward, which challenges one faces and how, and where one’s sense of self and life beyond the reading situation comes in. Based on the perspectives, we demonstrate that children have varied ways of being absorbed when reading fiction, and we reflect on the affordances of Q methodology as a suitable child-centered approach to studying the subjective experiences of reading.

### Absorbed reading

Narrative fiction experiences are relatively widely researched in studies with adult participants, particularly regarding literary texts and the effects of specific linguistic structures (Kuiken and Jacobs, 2021). One of the field’s strongest endeavors in recent years has been the systematic study of readerly absorption (Hakemulder et al., 2017; Kuijpers et al., 2021; Kuijpers, 2022) and absorption-like states (Kuiken and Douglas, 2017; Kuiken et al., 2022). Competing with a number of close conceptual relatives such as narrative engagement (Busselle and Bilandzic, 2009), flow (Thissen et al., 2018), immersion (Jacobs and Lüdtke, 2017) and so forth (for reviews see Kuijpers et al., 2021; Pianzola, 2021), absorption is an increasingly dominant multidimensional construct for describing sustained, intrinsically rewarding fiction reading.

The central questionnaire for measuring absorption, the Storyworld Absorption Scale (SWAS; Kuijpers et al., 2014), is agnostic to literary merit. Its components can in theory also be used for exploring experiences of the “formulaic books, series books or what *seem* to be simpler texts” (Wilhelm, 2016; our italics) that many children choose to engage with in their spare time. The main components of absorption (and related constructs) have been abstracted by Kuijpers et al. (2021) as follows: ‘attention’ (e.g., resistance to distraction, altered sense of time); ‘mental imagery’ (mainly but *not only* visualization); ‘emotional engagement’ (e.g., sympathy, empathy for character); ‘transportation’ (experiences of having entered the story world). These components also correspond to the four subscales of the SWAS (Kuijpers et al., 2014), where ‘attention’ has been identified as a possible predictor of the remaining three dimensions (Kuijpers, 2022).

The literature on absorption largely creates the impression that there is one universal way of being absorbed. As Kuiken et al. (2022, p. 2) note, “empirically substantiating differences between two or more theoretically independent modes of deeply engaged literary reading within the same population has not (…) been attempted.” As a start to such work, Kuiken and Douglas (2017; Kuiken et al., 2022) have developed the Absorption-like States Questionnaire (ASQ). This tool captures two different routes to “reflective and creative” absorption: expressive enactment (ASQ EE) and integrative comprehension (ASQ IC). These routes differ e.g., with regard to ‘peri-personal vs. extra-personal space’ (in expressive enactment/EE the story world feels within reach; in integrative comprehension/IC it is perceived from a distal vantage point), self-other relations (in EE one merges with a character through ‘pre-enactive empathy;’ in IC one assesses characters’ states from the outside via ‘cognitive perspective-taking’), or verisimilitude (in EE one’s self is implicated through reminding with of one’s own life; in IC one relies on knowledge of the world and people more generally).

The latter distinctions are an important move toward recognizing plurality in readerly absorption. Unlike other measures, the ASQ covers nuances in notional position vis-à-vis story world and characters and, crucially, discriminative awareness of one’s self beyond the moment of reading. The latter is unusual as one’s self is assumed largely “lost” in reading as per the absorption literature (Kuijpers et al., 2021). The different facets of absorption are indeed useful in interpreting various types of reading data across age groups and text genres, from experiments (Mangen and Kuiken, 2014) to online reviews (Rebora et al., 2021), although the Absorption-like States Questionnaire specifically was developed with “*difficult* linguistic structures” (Kuiken et al., 2022; our italics) in mind. However, the questionnaires as a tool, and their often intricate wordings (e.g., “my feelings were as ‘close’ for me as they were for the character whose point of view was being presented there;” Kuiken et al., 2022), may not be suitable for child-centered research.

### Child-centered approaches to reading experience

Heeding contemporary calls for supporting children’s own voices in research and society (Christensen and James, 2017), children’s subjectivity in and around reading needs to be studied with tools more open-ended than traditional questionnaires, also because reading styles evolve throughout one’s entire lifespan (Charlton et al., 2004) but most dynamically during childhood (Compton-Lilly, 2016). A step in this direction was recently taken by McGeown et al. (2020) who chose to abandon the established Motivation for Reading Questionnaire (MRQ; Wigfield and Guthrie, 1997; Schiefele and Löweke, 2018) in their exploration of children’s reading motivations and concomitant choices among book, magazine, newspaper, and other formats. Instead of having children rate a battery of items effectively testing them for their levels of extrinsic (e.g., “I hate it when others read better than me.”) vs. intrinsic motivation (e.g., “I enjoy a long, involved story or fiction book.”), the authors conducted qualitative interviews in which children aged 9–11 also acted as co-participant researchers. An alternative set of reading motivations was identified that partly overlapped with those recognized by the MRQ (e.g., ‘social motivation,’ ‘skill development’) and partly exceeded them (e.g., ‘relax,’ ‘engage with the familiar’); some MRQ subscales (e.g., ‘reading for school grades’) were in turn not covered.

Studies of children’s reading motivation such as those above cover a wide spectrum of reading materials beyond fiction; they also focus more on reading behaviors than inner experiences proper. Once we zoom in on fiction, the more complex descriptions of meetings between readers and texts tend to be based on field observations rather than interviews (Wolf and Heath, 1992; Mackey, 2007; Lenters, 2018). Field observations inevitably center on the overt interactions (child–child; child–adult) that take place around reading. Although capturing moments of emotional engagement, self-other connection, and so forth, they cannot account in detail for those moments’ inner subjective feel. Moreover, certain aspects of absorbed reading, e.g., the sheer experience of paying attention or that of having temporarily relocated into a story world, may escape verbal exchange or even non-verbal expression. Sipe (2000, p. 267) conducted extensive observations of first- and second-graders’ (6–8 years) responses to classroom read-alouds and found some, though relatively sparse, explicit references to such experiences, concluding that “the best indication of such an experience is not verbal response, but silence.”

Interviews on selected aspects of children’s fiction reading are not uncommon *per se*; they have been used to complement observations (e.g., Mackey, 2007), quantitative surveys (e.g., Merga, 2017) or think-aloud responses to texts (e.g., Smith, 1991). Wilhelm (2016) has conducted an interview study exploring children’s self-chosen fiction reads in terms of a more nuanced range of subjective states closely related to absorption and motivation. Wilhelm conceptualizes these states as distinct ‘pleasures,’ having run four in-depth interview sessions over three years with a cohort of fiction-loving eighth-graders (13–14 years initially) and a cohort of secondary students who preferred “marginalized” genres specifically (romance, vampire stories, etc.). The following categories of ‘pleasure’ emerged across both cohorts: ‘immersive play’ (a sense of total engagement; a prerequisite of the remaining categories); ‘intellectual’ (figuring things out); social (reading as conduit to others or one’s self); ‘practical work’ (usefulness for other tasks); ‘inner work’ (actualizing one’s personal potential). All five categories were identified in all participants and Wilhelm argues for their recognition toward developing more child-centered pedagogies. Their relative weight was not compared across participants, however, to expose individual differences or distinct groups of children who share similar emphases. The latter was a key objective of our current study.

## Materials and methods

### Q methodology

In the current study, Q methodology was used to preserve the benefits of both quantitative inquiry and open-ended interviewing. Developed in the 1930s for the scientific study of subjectivity (Stephenson, 1936), Q methodology combines a philosophical framework (Q methodology), a data collection technique (Q sort), and a method of analysis (Q factor analysis). This distinctive set of psychometric, operational, and analytical principles provides a systematic and rigorous means to investigate shared perspectives (Stephenson, 1953; McKeown and Thomas, 2013). Q methodology works on two assumptions. Firstly, human subjectivity has a measurable internal structure that provides each individual with a frame of reference for understanding the world around them. This structure can be observed through an individual’s actions and modeled based on a systematic ordering of stimuli selected for this purpose. Secondly, the measurement must take place in such a way that participants can dispose of and interpret the meanings of the stimuli. When examining subjectivity, it is necessary to respect the internal frame of reference of the participant (Stephenson, 1953).

Participants in a Q study are presented with a set of cards with authentic experience labels which they are invited to sort. These items – typically verbal, sometimes pictorial (Ellingsen et al., 2014) – are generated either from preliminary qualitative research or from literature reviews and other relevant sources. Our Q sessions consisted of two distinct but interconnected Q studies, both based on the same preliminary research (Kuzmičová et al., 2022; details below): Q Study 1, reported in Supa et al. (Submitted), and Q Study 2, focal to the present article. Q Study 1 used pictorial items which were modeled on children’s statements and video stills of their behavior; participants sorted 19 cards depicting different ways of “being with” a story in terms of modalities/activities (e.g., reading; being read to; listening to audio; watching film; playing and playing out; creating; thinking) and situations (postures; degrees of body stability/mobility; degrees of privacy; being tucked in/away; etc.). In the focal Q Study 2, participants sorted 24 verbal statements expressing different inner states and expectations relating to fiction reading, specifically.

**FIGURE 1:**
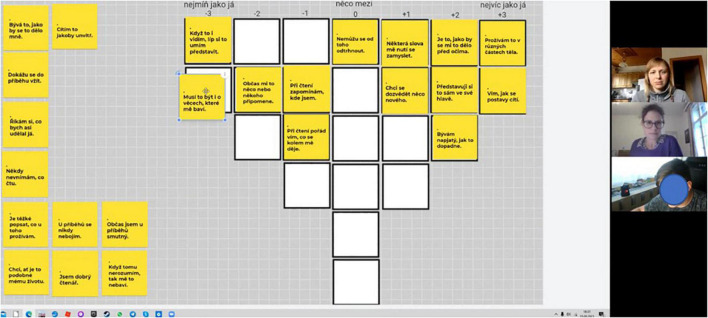
Still from online research session: the sorting interface **(left)**, researchers and participant **(right)**.

Once acquainted with the items, participants are guided by successive steps to compare them with each other and sort them onto a bell-shaped (quasinormal) grid. A typical instruction is “Sort the cards according to what is most like (+3) and most unlike (–3) your everyday feelings” (McKeown and Thomas, 2013; Ellingsen et al., 2014). Participants should arrive at their quasinormal distribution within the given grid so that at the extreme ends, there are only a few items, and toward the middle of the grid, the number of items increases (see Figure 1). The resulting Q sort reflects the participant’s subjective view of the topic. After sorting, an interview is conducted which focuses primarily on the participant’s understanding of items at the extreme ends, inviting the participant to explain the main rationale behind their Q sort. The post-sorting interview facilitates interpretation of the results and can also draw attention to differential understandings of an item between groups of participants.

In the last step, participants’ Q sorts are transformed into a raw data matrix and analyzed. The analysis consists of three sequentially applied statistical procedures: correlation, Q factor analysis, and the computation of factor scores (McKeown and Thomas, 2013). The correlation matrix provides a preview of relationships between individual participants. Above all, it serves as input for factor analysis that calculates the number of independent factors. Where traditional (“R”; Stephenson, 1936) factor analysis identifies correlations between variables across participants, Q factor analysis identifies correlations between participants across variables. Each factor represents a group of individual Q sorts that are highly correlated while being uncorrelated with the others. The factor loadings then express the degree to which a given Q sort is associated with each factor. A high positive factor loading indicates that a given participant shares that particular perspective with others, and conversely, a high negative factor loading indicates rejection of that perspective. Finally, factor scores – or *z*-scores – reflect the degree of agreement of individual statements with the identified factors. Thus, factors are usually called perspectives or viewpoints because they reveal shared views of participants (Brown, 2004). Q methodology does not seek to generalize findings beyond the given group of participants. The relevance of participants to the research question is crucial for their inclusion in the sample. Generalizations should be thought of in terms of the universe of subjectivity rather than in terms of the population’s characteristics (Brown et al., 2015, p. 534).

Q methodology research with children and adolescents has appeared in many disciplines including childhood and youth studies (Kerpelman et al., 2002; Metzger et al., 2016), psychology (Richards et al., 2007; Krause et al., 2021), and primary education (Tan et al., 2015). To our knowledge there have not been any Q studies conducted with children or adolescents regarding their reading experience, despite the method’s increasing acceptance in both compulsory education research (Lundberg et al., 2020) and audience research (Davis and Michelle, 2011). One Q study on reading (Levitt and Red Owl, 2013) investigated the attitudes, behaviors, and values of veteran teachers. Q methodology is inherently child-centered, aligning with voices asserting the need to study children’s lived experience and their subjective understanding of the world (Greene, 2006; Hughes, 2016). If designed with care and consideration, it works as an inclusive, respectful, and ethical methodology that challenges a one-size-fits-all approach “by hearing a range of voices, including those marginalized” and that decreases the risk of adult perspectives overshadowing those of children and youth in social research (Hughes, 2016, p. 63).

### Items for assessment (Q sample)

The selection of items to be presented to participants (Q sample) is a critical moment that fundamentally affects the outcome of a Q study. Care must be taken that key dimensions of subjectivity relevant to the research topic are represented within a relatively limited set of items; Q samples used with children normally range between 20 and 50 items (Ellingsen et al., 2014). The items in our focal study were gathered in preliminary research using creative focus groups and individual interviews (*n* = 19; *M*_*age*_ = 10.09), complemented with in-class observations of spontaneous literacy activities. This preliminary research, reported in Kuzmičová et al. (2022), facilitated participants’ introspection through bespoke physical props and game-like activities. Conversations about habitual book selections and experiences were combined with direct story exposure; the children not only talked about their favorite stories but also listened to pre-selected read-alouds and watched film snippets, using the props to share their immediate responses. The pivotal questions were how varied engagements with fictional stories (reading, watching, listening, playing, performing, creating, telling, and more) feel like from within one’s body; how one’s body is configured during the activities; what inner experiences are desired and typical and where one’s mind is felt to be (“here” in the room vs. “there” in the story world vs. “elsewhere”) when one engages with stories in different ways.

To arrive at the items used in our Q study, the interview and focus groups transcripts and observational notes from the preliminary research were first openly coded and inductively analyzed by the second author of this article, who drew on her expertise in children and youth’s lived media experience and learning (Neag and Supa, 2020; Ramsey et al., 2022). Throughout the process, importantly, this researcher remained naïve to any existing reading experience constructs related to absorption (Kuijpers et al., 2021; Kuiken et al., 2022), reading motivation (Wigfield and Guthrie, 1997), or children’s reading ‘pleasures’ (Wilhelm, 2016) as reviewed above. Five inductive categories relevant to absorbed reading emerged from the preliminary analysis and interpretation, to which the researcher gave experience-centered labels: ‘living it,’ ‘imagining it,’ ‘feeling it,’ ‘reading it,’ and ‘experiencing closeness/otherness.’ These categories collectively represented shared patterns concerning a focal phenomenon, as this research phase focused on similarities rather than differences. For example, the ‘experiencing closeness/otherness’ category included statements on stories’ different degrees of closeness to one’s life and interests and on the life-to-text and text-to-life connections such personal relevance, or lack thereof, might entail (Kuzmičová and Bálint, 2019; Kuzmičová and Cremin, 2022).

As the codes under each category were easily linked back to children’s direct quotes, these quotes were then used to develop representative statements for the five categories. The statements in our Q research are therefore based very closely (even verbatim) on children’s authentic statements extracted from the preliminary research. Although some of the statements could possibly fall within more than one category, the aim was to place them under a single dominant category, which then allowed reducing our initial, much larger sample to 22 statements while seeking relative balance across the categories (McKeown and Thomas, 2013). Following pilot sessions with three children and discussions with the remaining authors, some statement wordings were slightly changed for accessibility and clarity, and two new items were added that were also based on the preliminary data (#18, #22). Thus we arrived at the final 24 statements.

The inductive categories served solely for the purposes of generating our Q sample in a bottom-up fashion and should not be thought of as an analytical tool in interpreting our data. Links with existing theoretical constructs, which nevertheless proved significant (see Table 1), were purposefully explored by the first author only after the Q sample had been finalized. The statements were in Czech, the local language where the study took place. Our own English translations are provided throughout this article. These are *ad hoc* translations for the purposes of the current report that draw on our experience with designing reader response questionnaire items (Kuzmičová et al., 2017; Magyari et al., 2020) and child-centered research in English-speaking environments (Woodfall and Zezulkova, 2016; Kuzmičová and Cremin, 2022); they have not been piloted for research in English.

**TABLE 1:** Final Q sample with inductive categories and subscales of the SWAS, ASQ EE/IC, MRQ (where supplementary) and reading ‘pleasures.’

#	Statement	Inductive category	Subscale (questionnaire)	Reading ‘pleasure’
1	It’s as if the same things were happening to me.	Living it	Pre-enactive empathy (ASQ EE)	
2	Stories make me feel things in different parts of my body.	Living it	*Mental imagery* (SWAS); *Pre-enactive empathy* + *Peri-personal space* (ASQ EE)	
3	I forget where I am when I read.	Living it	Attention + *Transportation* (SWAS)	
4	I feel connected with what is happening in the story.	Living it	Emotional engagement (SWAS); *Peri-personal space* + Pre-enactive empathy (ASQ EE)	Immersive play
5	I can’t stop reading.	Living it	*Transportation*	Immersive play
6	It’s as if it were happening in front of my eyes.	Imagining it	Mental imagery (SWAS); Extra-personal space (ASQ IC)	
7	I wonder what I would do.	Imagining it		Inner work
8	It’s easier to imagine if I can see it too.	Imagining it	Mental imagery* (SWAS)	
9	I imagine it in my head.	Imagining it	Mental imagery (SWAS)	
10	I feel things happening inside me.	Feeling it	*Pre-enactive empathy* (ASQ EE)	
11	Sometimes I am sad.	Feeling it	*Emotional engagement* (SWAS)	
12	I know how the characters feel.	Feeling it	Emotional engagement (SWAS); Pre-enactive empathy (ASQ EE); Cognitive perspective-taking (ASQ IC)	Social
13	I am often tense about what will happen next.	Feeling it	*Reading involvement* (MRQ)	*Immersive play*
14	It’s hard to describe what it’s like for me.	Feeling it		
15	I am never scared.	Feeling it	Emotional engagement* (SWAS)	Immersive play*
16	I am not always following what I am reading.	Reading it	Attention* (SWAS)	Immersive play*
17	I am a good reader.	Reading it	Efficacy (MRQ)	
18	Some words make me think.	Reading it		Intellectual
19	I always know what is happening around me.	Reading it	Attention* (SWAS)	Immersive play*
20	I want it to resemble my life.	Closeness/ otherness	*Self-implicating givenness* (ASQ EE)	Social
21	When I don’t understand it, I don’t enjoy it.	Closeness/ otherness	Reading work avoidance (MRQ)	Intellectual*
22	It must also be about things I like.	Closeness/ otherness	Curiosity (MRQ)	
23	I want to learn something new.	Closeness/ otherness	Curiosity (MRQ)	Intellectual
24	Sometimes it reminds me of something or someone.	Closeness/ otherness	*Self-implicating givenness* (ASQ EE); Affective realism + Character realism (ASQ IC)	Social

Reading ‘pleasures’ appear where wording adheres closely to data cited by Wilhelm (2016). Asterisk indicates reverse perspective. Italics indicate weaker correspondence, i.e., our wording is significantly narrower in scope than given questionnaire item(s) (e.g., #16 vs. SWAS item EE5) or differs in modality (e.g., #19 vs. ASQ EE items MM2-3).

### Participants (P set)

The participants were 28 children aged 9–12 years (*M* = 10.54), the conventional P set (“P” = participant) size in Q methodology being 20–60 participants (Brown, 1993; Watts and Stenner, 2005). Nine to twelve years is for many a crucial stage in reading development (Compton-Lilly, 2007; Torppa et al., 2020) yet understudied with regard to subjective experiences of the reading process. At the age of nine, reading in a technical sense has typically been acquired by Czech L1 learners as Czech has a consistent orthography and is relatively easy to decode (Caravolas et al., 2013). This is a developmental stage when parents often step away from shared reading, if practiced at all (Merga, 2017; Scholastic, 2018).

Whilst aware that cognitive development cannot be reduced to age alone, our chosen age bracket normally falls under middle and/or late childhood in developmental accounts. Toward the end of late childhood, corresponding to our upper limit of twelve years, children typically begin reaching adult levels of diverse executive functions after which cognitive changes slow down (Buttelmann and Karbach, 2017). Research on maturation in the domains of psychomotor function, attention, working memory, and visual learning (ages 10–18 years) revealed that improvements in speed and accuracy occurred at the greatest magnitude in ages ten to twelve specifically (Cromer et al., 2015). Similarly, the development of social cognition, which is instrumental to – and reportedly fostered by Mar and Oatley (2008) – the processing of narrative fiction, peaks around the transition from late childhood to adolescence (Mill et al., 2014). This suggests a sensitive and dynamic developmental period during which variations in conscious reading experience deserve special attention.

Our study took place in Czechia. A half of the children had participated in the preliminary research (*n* = 8 focus groups; *n* = 6 interviews) and a half were recruited through snowball sampling using the participating children’s and their parents’ social networks (Browne, 2005; Johnston et al., 2010). We used a strategic approach to participant recruitment, looking for a diverse group of participants who would be “likely to express a particularly interesting or pivotal point of view” (Watts and Stenner, 2012, p. 71). The final P set was balanced for gender (14 males, 14 females) and roughly also age (9–12 years) in combination. Among the male participants, there were three 9-year-olds, three 10-year-olds, five 11-year-olds, and three 12-year-olds. Among the female participants, there were three 9-year-olds, four 10-year-olds, four 11-year-olds, and three 12-year-olds.

The participants attended varied schools (public and private, traditional and Montessori), had parents with diverse education levels (from secondary vocational to postgraduate), and lived in urban and rural areas across the country. However, we did not control for social disadvantages (e.g., minority background, non-traditional family, low-income household, etc.); future research could be more sensitive to variation in this matter. In addition to demographic criteria, crucial to our sampling and recruitment strategy was that each participant had independent fiction reading experience and we also took care to include a relatively wide spectrum of overall attitudes to reading (based on the children’s or gatekeepers’ – parents’, teachers’ – perceptions). Thus, we ensured that the P set would be relevant and sufficiently heterogeneous (Watts and Stenner, 2012).

Written informed consent was obtained from parents and repeated oral informed consent was provided by the participants. Ethical standards were met specific to participatory research with children (aged 12 and below), while addressing power dynamics and applying strategies to support children in expressing their authentic voices (Montreuil et al., 2021). The research was approved by the Ethics Committee of the Faculty of Arts, Charles University, clearance number UKFF/151685/2021.

### Data collection (Q sorting)

Data was collected remotely via the Zoom Meetings software as the research took place in early stages of the COVID-19 pandemic. Within each Zoom session participants completed the two sorts, Q Study 1 and Q Study 2, consecutively using the Jamboard interactive whiteboard in Google Workspace, while being guided by a moderator and co-moderator. In the focal Q Study 2, the statements were shown as virtual post-it notes that could be moved around and flexibly rearranged on the sorting grid (Figure 1). The decision to involve a co-moderator was motivated by the unusual remote interviewing situation; the co-moderator explained technicalities (screen sharing, virtual card sorting) to the participants and was prepared to serve as backup should the moderator be disconnected unexpectedly. The post-sorting interviews were led by the moderator.

Participants were invited to follow their own subjective ideas of the different inner states captured by the verbal statements, and to sort them accordingly using a bell-shaped sorting grid with a 7-point scale of similarity from *Least like me* (–3) to *Most like me* (+3). At this point they were accustomed to the research activity, having completed the pictorial sort and post-sorting interview of Q Study 1. Participants’ occasional questions regarding the meaning of a statement were answered in such a way that interpretation would be constrained as little as possible. Participants’ individual interpretations of the verbal statements were explored in the post-sorting interviews. The moderators asked especially about motivations behind extreme card placements (i.e., ranking positions –3, –2; +2, +3) and about examples of personal situations and experiences captured by these statements. Cards that were more centrally (i.e., neutrally) placed within the sorting grid were also discussed as time permitted, especially when they seemed to suggest interesting relationships to the extremities. Participants could elaborate freely, and it was made clear to them that no detail of their experience was irrelevant. Statement #17, “I am a good reader,” was always discussed irrespective of placement. Focus was maintained on individual, silent, volitional reading of book-length fiction, although some participants also mentioned experiences of communal reading aloud in both home and school settings, and of stories in other genres and media.

The meetings lasted up to sixty minutes comprising both Q sorts and were video recorded. The participants shared their screen while sorting and in all but one case agreed to having their inbuilt camera switched on throughout the process. The moderator and co-moderator were visible at all points of interaction. Sorting was self-paced. Participants were encouraged to take breaks when needed and aware that they can terminate the session at any time. They could ask questions at the end of the session. Some children wanted to know more about the research aim and purpose, others expressed satisfaction with being able to reflect and/or being listened to, and a few asked for feedback regarding their “performance.” A vast majority spontaneously commented that they enjoyed the sorting and post-sorting discussion.

### Analyses (Q factor, qualitative analysis)

The sorts were subjected to by-person factor analysis by the third author, using Ken-Q Analysis v1.0.6 and employing the principal component method (PCA). In the focal Q Study 2, three factors were selected for further analysis based on the following criteria (see Watts and Stenner, 2005; McKeown and Thomas, 2013): (1) eigenvalue size greater than one; (2) at least two Q sorts have statistically significant loadings at 0.05 for a given factor; (3) the minimum size of explained variance is 10 percent. Inter-factor correlations ranged from –0.45 to 0.43, without reaching statistical significance at *p* < 0.01, suggesting that distinct perspectives had been extracted. These were subsequently rotated using the varimax procedure and Factors 1 and 3 were manually rotated by –5 degrees. This procedure improved the distribution of participants among factors. One of the factors (F1; “F” = factor) was bipolar, i.e., featured two groups of participants whose sorts were statistically inverse to each other. Two sorts (P21, P28) were removed; P21 did not load significantly onto any factor and P28 was inverse to F3. The last step involved splitting the bipolar F1 into two perspectives: F1a and F1b.

Selected factors accounted for 48 percent of the total variance. None of the final factors significantly dominate in terms of the number of sorts and variance explained: F1a and F1b comprised three and five participants respectively (14% variance), F2 comprised nine participants (18% variance), and F3 likewise comprised nine participants (16% variance). A composite Q sort was generated for each factor representing the sorting of a hypothetical participant matching the given factor to the maximum extent possible, i.e., having a hundred percent factor loading (Tables 2–5). Difference scores were likewise calculated which express the magnitude of the difference between the *z*-scores of a particular statement for any two factors. If this difference is statistically significant, the item is a distinguishing statement that splits two (or more) perspectives. If a statement’s *z*-scores do not statistically differ between any pair of factors, it is a consensus statement. All composite Q sorts are summarized in the factor array in Table 6 where *z*-scores and distinguishing/consensus status are also reported for each statement.

**TABLE 2:** Composite Q sort – *Growth* (F1a).

–3	–2	–1	0	+1	+2	+3
#8** < It’s easier to imagine if I can see it too.	#10* < I feel things happening inside me.	#3* I forget where I am when I read.	#7 I wonder what I would do.	#6 It’s as if it were happening in front of my eyes.	#1** > It’s as if the same things were happening to me.	**#9** I imagine it in my head.
**#21** When I don’t understand it, I don’t enjoy it.	#22* < It must also be about things I like.	#11 Sometimes I am sad.	#13 I am often tense about what will happen next.	#23 I want to learn something new.	#4** > I feel connected with what is happening in the story.	#2** > Stories make me feel things in different parts of my body.
	#24 Sometimes it reminds me of something or someone.	#16 I am not always following what I am reading.	#14 It’s hard to describe what it’s like for me.	#5 I can’t stop reading.	**#17** I know how the characters feel.	
		#19 I always know what is happening around me.	#15 I am never scared.	#17 I am a good reader.		
			#18 Some words make me think.			
			#20 I want it to resemble my life.			

Bold # marks items that are key in both quantitative (extreme and/or statistically distinguishing rank) and qualitative terms (interview analysis). Distinguishing items are marked with * (*p* < 0.05) or ** (*p* < 0.01) and < (*z*-score lower than remaining factors) or >(*z*-score higher than remaining factors). For key items, background colors refer to inductive categories: ‘living it’ – yellow, ‘imagining it’ – pink, ‘feeling it’ – green, ‘reading it’ – purple, ‘experiencing closeness/otherness’ – orange.

**TABLE 3:** Composite Q sort – *Confirmation* (F1b).

–3	–2	–1	0	+1	+2	+3
#3 I forget where I am when I read.	**#1** It’s as if the same things were happening to me.	#5** < I can’t stop reading.	#6 It’s as if it were happening in front of my eyes.	#21 When I don’t understand it, I don’t enjoy it.	**#9** I imagine it in my head.	#8** > It’s easier to imagine if I can see it too.
#2** < Stories make me feel things in different parts of my body.	#4** < I feel connected with what is happening in the story.	#10 I feel things happening inside me.	#11 Sometimes I am sad.	#7 I wonder what I would do.	#12** > I always know what is happening around me.	**#13** I am often tense about what will happen next.
	#17* < I know how the characters feel.	#20 I want it to resemble my life.	#17 I am a good reader.	#14 It’s hard to describe what it’s like for me.	#22** > It must also be about things I like.	
		#15 I am never scared.	#18 Some words make me think.	#16 I am not always following what I am reading.		
			#23 I want to learn something new.			
			#24 Sometimes it reminds me of something or someone.			

Bold # marks items that are key in both quantitative (extreme and/or statistically distinguishing rank) and qualitative terms (interview analysis). Distinguishing items are marked with * (*p* < 0.05) or ** (*p* < 0.01) and < (*z*-score lower than remaining factors) or >(*z*-score higher than remaining factors). For key items, background colors refer to inductive categories: ‘living it’ – yellow, ‘imagining it’ – pink, ‘feeling it’ – green, ‘reading it’ – purple, ‘experiencing closeness/otherness’ – orange.

**TABLE 4:** Composite Q sort – *Attachment* (F2).

–3	–2	–1	0	+1	+2	+3
#3 I forget where I am when I read.	**#1** It’s as if the same things were happening to me.	#2 Stories make me feel things in different parts of my body.	#14* < It’s hard to describe what it’s like for me.	#11** > Sometimes I am sad.	**#4** I feel connected with what is happening in the story.	#9* > I imagine it in my head.
**#15** I am never scared.	#8** It’s easier to imagine if I can see it too.	#19 I always know what is happening around me.	#21* When I don’t understand it, I don’t enjoy it.	#12 I know how the characters feel.	**#5** I can’t stop reading.	**#13** I am often tense about what will happen next.
	#16* < I’m not always following what I’m reading.	#23 I want to learn something new.	#10 I feel things happening inside me.	#7 I wonder what I would do.	#6* > It’s as if it were happening in front of my eyes.	
		#24 Sometimes it reminds me of something or someone.	#18 Some words make me think.	#17 I am a good reader.		
			#20 I want it to resemble my life.			
			#22 It must also be about things I like.			

Bold # marks items that are key in both quantitative (extreme and/or statistically distinguishing rank) and qualitative terms (interview analysis). Distinguishing items are marked with * (*p* < 0.05) or ** (*p* < 0.01) and <(*z*-score lower than remaining factors) or >(*z*-score higher than remaining factors). For key items, background colors refer to inductive categories: ‘living it’ – yellow, ‘imagining it’ – pink, ‘feeling it’ – green, ‘reading it’ – purple, ‘experiencing closeness/otherness’ – orange.

**TABLE 5:** Composite Q sort – *Mental Shift* (F3)

–3	–2	–1	0	+1	+2	+3
#19** < I always know what is happening around me.	#18** < Some words make me think.	#6** < It’s as if it were happening in front of my eyes.	#10 I feel things happening inside me.	#8** It’s easier to imagine if I can see it too.	#4 I feel connected with what is happening in the story.	#3** > I forget where I am when I read.
#15 I am never scared.	#2 Stories make me feel things in different parts of my body.	#1 It’s as if the same things were happening to me.	#11 Sometimes I am sad.	#9* < I imagine it in my head. .	**#16** I am not always following what I am reading.	#5** > I can’t stop reading.
	**#21** When I don’t understand it, I don’t enjoy it.	#20 I want it to resemble my life.	#17 I know how the characters feel.	#7 I wonder what I would do.	**#13** I am often tense about what will happen next.	
		#22 It must also be about things I like.	#14 It’s hard to describe what it’s like for me.	#23 I want to learn something new.		
			#17 I am a good reader.			
			#24 Sometimes it reminds me of something or someone.			

Bold # marks items that are key in both quantitative (extreme and/or statistically distinguishing rank) and qualitative terms (interview analysis). Distinguishing items are marked with * (*p* < 0.05) or ** (*p* < 0.01) and <(*z*-score lower than remaining factors) or >(*z*-score higher than remaining factors). For key items, background colors refer to inductive categories: ‘living it’ – yellow, ‘imagining it’ – pink, ‘feeling it’ – green, ‘reading it’ – purple, ‘experiencing closeness/otherness’ – orange.

**TABLE 6:** Factor array.

#	Statement	F1a		F1b		F2		F3	
		Sort	Zsc	Sort	Zsc	Sort	Zsc	Sort	Zsc
1	It’s as if the same things were happening to me.	2**	1.1	–2	–1.1	–2	–0.8	–1	–1.0
2	Stories make me feel things in different parts of my body.	3**	1.8	–3**	–2.2	–1	–0.7	–2	–1.1
3	I forget where I am when I read.	–1*	–0.5	–3	–1.4	–3	–1.3	3**	1.7
4	I feel connected with what is happening in the story.	2	1.1	–2**	–1.0	2	1.4	2	1.5
5	I can’t stop reading.	1	0.6	–1**	–0.6	2	1.0	3**	1.9
6	It’s as if it were happening in front of my eyes.	1	0.5	0	0.1	2*	1.3	–1**	–0.9
7	*I wonder what I would do.†*	0	–0.3	1	0.3	1	0.3	1	0.4
8	It’s easier to imagine if I can see it too.	–3**	–2.0	3**	1.8	–2**	–1.1	1**	0.9
9	I imagine it in my head.	3	1.7	2	1.2	3*	2.3	1**	0.5
10	I feel things happening inside me.	–2*	–1.2	–1	–0.5	0	0.2	0	0.0
11	Sometimes I am sad.	–1	–0.7	0	–0.3	1**	0.6	0	–0.1
12	I know how the characters feel.	2	1.0	–2*	–0.6	1	0.5	0	0.1
13	I am often tense about what will happen next.	0	0.5	3	1.7	3	1.6	2	1.0
14	It’s hard to describe what it’s like for me.	0	0.4	1	0.4	0*	–0.4	0	0.3
15	I am never scared.	0	–0.3	–1	–0.5	–3	–1.5	–3	–1.3
16	I am not always following what I am reading.	–1	–0.5	1	0.2	–2*	–1.2	2**	1.1
17	*I am a good reader.†*	1	0.5	0	–0.1	1	0.3	0	–0.2
18	Some words make me think.	0	0.1	0	0.2	0	0.2	–2**	–1.2
19	I always know what is happening around me.	–1	–0.5	2**	0.9	–1	–0.7	–3**	–1.6
20	*I want it to resemble my life.††*	0	0.0	–1	–0.5	0	–0.3	–1	–0.5
21	When I don’t understand it, I don’t enjoy it.	–3	–1.6	1*	0.8	0*	0.1	–2	–1.0
22	It must also be about things I like.	–2*	–1.5	2**	1.7	0	–0.4	–1	–0.7
23	I want to learn something new.	1	0.6	0	–0.3	–1	–0.5	1	0.6
24	*Sometimes it reminds me of something or someone.*	–2	–0.8	0	–0.3	–1	–0.8	0	–0.4

Ranks of distinguishing statements are shown in bold (*p < 0.05, **p < 0.01); consensus statements in italics (^†^ – non-significant at *p* > 0.01, ^††^ – non-significant at *p* > 0.05).

The composite Q sorts and factor array were instrumental in the qualitative phase of data analysis. Once the Q factor analysis was completed, the first and second author divided the transcripts of the post-sorting interviews of retained participants (*n* = 26) according to the three factors and four perspectives. Next, the transcripts and quantitative results were inductively analyzed in tandem, first within each group and then in relation to other perspectives. For these inductive-deductive processes, reflexive thematic analysis (Braun and Clarke, 2006) was used as a framework. The transcripts were independently read and re-read in conjunction with the composite Q sorts by the first two authors before the whole research team jointly discussed first impressions and moved to systematic coding of the qualitative data (Hayfield and Wood, 2019). Yet instead of conceptualizing themes through the coding, we conceptualized perspectives by identifying “patterns of shared meaning” consisting of the most prevalent codes (Braun and Clarke, 2019, p. 592). These patterns both characterized the reading engagement of children within a given perspective, as well as distinguished them from children in the other groups. After defining the essence of individual perspectives and naming them, final additional adjustments were made during the writing process. The outcome is presented in the next section.

## Results

The emergent perspectives on absorbed fiction reading reflected by the factors were interpreted and labeled as follows (in ascending order by *n* participants): *Growth* (F1a), *Confirmation* (F1b), *Attachment* (F2), *Mental Shift* (F3). These four perspectives are described in the remainder of this section. Each description opens with a summary, then follows two non-exclusive headings: *Reading* (what feeds into and happens in the reading situation) and *Beyond reading* (how reading relates to life beyond the reading situation). Relevant Q items are shown in brackets, represented by statement # and ranking. Where a statement’s ranking is statistically distinguishing for the perspective, degrees of significance are marked by one (*p* < 0.05) or two (*p* < 0.01) asterisks, respectively. For example, “(#8 –3^**^; #9 + 3)” means that a specific point in our interpretation is reflected in a given group’s distinguishingly low (*p* < 0.01) ranking of statement #8 and very high (but not statistically distinguishing) ranking of statement #9. This information is also provided in Tables 2–6. All direct quotes from participants’ post-sorting comments, translated into English by the authors, are accompanied by participant ID and participant’s gender (m x f) and age (9–12). For example, “(P23, f9)” stands for Participant 23, female, 9 years old. Each participant is quoted at least once. Throughout this section, we deliberately refrain from using the technical nomenclature linked to absorption and similar constructs, in keeping with the inductive nature of our interpretation of the perspectives. Composite Q sorts are provided, visually coded to show which statements are key in quantitative (extreme and statistically distinguishing ranks) as well as qualitative terms (Tables 2–5).

### Growth (F1a)

#### Summary

Under the *Growth* perspective, written fiction of the most varied kinds is experienced holistically as children think through scenarios, imagine story worlds, and sympathize with characters while also adopting their bodily sensations. For children sharing this perspective, reading is in a close and reciprocal relationship with living, learning, and growing as a person. The main focus to being absorbed in stories is on one’s own reflective processes during reading and, crucially, beyond the reading situation.

#### Reading

Reflective processes are constantly at work in the *Growth* perspective and children are open to tackling any interpretive hurdles or unfamiliar themes (statement #22 ranked –2^**^; statement #21 ranked –2). Written stories are expressly acknowledged in their power to develop one’s imagining, thinking and knowing, and most are therefore potentially of interest, however challenging: “I wasn’t so much into WWII because it was just terrible (…) but I wanted to know how things went in the end” (P23, f9); “so I go on and try to look things up to understand better instead of just saying this is not for me” (P13, f12).

Varied forms and dimensions of absorption combine here in a rounded reading experience. This is the only perspective wherein children attest to vicariously adopting characters’ movements and inner embodied sensations (#2 + 3^**^; #1 + 2^**^; #4 + 2): “like when it says someone’s scratched the blackboard with his fingernails then I feel – it comes to me just, like it’s me (…) who’s doing it” (P13, f12); “it’s actually me holding the sword swinging it in the air” (P19, m12). Sometimes this tendency translates into reflexive real-world actions when a child checks their own body based on the text: “when there was a fight and someone lost a tooth, at that moment I put my hand to my mouth (…) when a character gets stabbed in the shoulder, I check my shoulder just in case” (P19, m12). At the same time, directly adopting characters’ experiences does *not* extend beyond bodily sensations, e.g., to notional vantage points. Rather, multiple vantage points are often creatively combined (#9 + 3; #6 + 1): “it’s as if I cloned myself and while I’m looking at me watching or taking part in things, this other me is doing those things but not seeing them through her eyes” (P13, f12).

The decoupling from characters is even more pronounced in terms of emotions and cognitive states. Children agree that their understanding is more a matter of conscious reflection (#12 + 2), a process of coming to know unexpected things which they so enjoy (#22 –2*; #21 –2; #23 + 1), rather than immediate empathic feeling (#10 –2*). They report assessing characters’ inner states “still being myself” (P23, f9), a buddy having “deep, deep sympathy” (P13, f12): “there’s a great deal of me working to live it through with (the character) (…) help them with the sadness a little bit” (P19, m12). Potentially unsettling events such as death are likewise reflected on analytically, in terms of their moral implications and their impact on other characters: “and then he kills this guy and I always get real angry, like why did he have to do it if he knew he was someone’s friend” (P23, f9). This reflective stance is maintained even when one becomes reminded, life-to-text, of similar events from one’s own past: “I know what it’s like when your best friend dies, it’s happened to me” (P23, f9).

#### Beyond reading

Stories in their written, non-pictorial form exclusively (#8 –3^**^; #9 + 3), fulfill an irreplaceable role in one’s personal growth and everyday life. Children knowingly use books for expanding their horizons beyond the moment of reading (#22 –2^**^; #21 –2; #23 + 1) and can spend hours, even months, reflecting on what they have read: “then I think of all sorts of alternative solutions (…) so I make the story last longer so to speak, it can be several months even” (P19, m12). Searches for additional relevant information commonly accompany this prolonged reflection. The insights thus encountered and worked through can exceed conventional reality boundaries as children show readiness to embrace alternative visions of the physical world or their potential selves: “I’m really a great optimist in this respect, I figure these things might happen, there might be wizards, there might be people with special skills (…) I dunno like people who have two mouths” (P13, f12).

Text-to-life transfers become manifest not only in thinking but also in overt behaviors. One child engages in reenacting physical actions from stories (#2 + 3^**^), then integrates her memories of these episodes with further reading: “I can make all these movements I’d make if I were there, I run, I climb trees and so on (…) it’s really nice because it makes me part of the story as it were” (P23, f9). For another (P13, f12), physical behaviors transfer text-to-life in the form of “like a new sport” or creative pastimes (figure drawing, modeling) that she picks up from characters. Children often vocalize their story-related thoughts for themselves both during and after reading as they literally engage in dialogue with the text “as if the story was happening in my brain and in my heart also” (P23, f9).

### Confirmation (F1b)

#### Summary

Under the *Confirmation* perspective, written stories must fall within one’s preferred subjects, genres and plotlines, and should ideally be illustrated and suspenseful, for moments of absorption to occur in the first place. Emphasis is on the text selected through these criteria which in turn are understood as its objective features rather than as a matter of subjective attitude. Reading is mostly about receiving confirmation in the given moment of one’s familiar ways of reading and living. Suspense is a prominent driver of reading and mental imagery a key sign of being absorbed.

#### Reading

Children sharing the *Confirmation* perspective prefer to stay within their comfort zone rather than wanting to be challenged by what they read (statement #21 ranked + 1*). Stories must conform to their predefined genre preferences and deal with subjects they find inherently interesting also in everyday life (#22 + 2*). Overall, these children show a heavily subject-based, even factual way of thinking about fiction reading. Instances of not understanding a text are defined as missing “what it’s about” (P4, m11), rather than grappling with narrative sequence or characters’ motivations; children also spontaneously name subjects that bore them: “say when there’s old historical buildings then I don’t like it” (P3, f12).

Above all, stories are expected to be suspenseful (#13 + 3). This makes certain parts of texts, such as beginnings, stand out as less entertaining because they try one’s patience; the same impatient tendency also surfaces when comprehension difficulties come up (#21 + 1*): “for me it wasn’t fun till someplace in the middle of the book, I mean the beginnings are – I always want the tension right away” (P25, m12); “rereading it is not exactly fun but I want to understand it a little bit” (P20, m11). Reading is abandoned and passages skipped at any point (#5 –1^**^) if a text does not fulfill the desired criteria and confirms the child in their reading endeavor: “when I’m bored, I skip the bit (…) when it’s not interesting I can quit easily” (P3, f12). Illustrations are another desired feature because they support one’s visual imaging (#8 + 2^**^): “they show me what I should imagine – and how” (P5, m9).

However, when all the above criteria are met, moments of focused fiction reading still tend to be relatively fleeting as one always remains aware of one’s surroundings (#19 + 2^**^; #5 –1^**^; #3 –3 understood as low ‘attention,’ see section “Discussion”). Immediate access to the story world or characters is not particularly lasting or robust, and even visual images, though important to the overall experience (#9 + 2), appear distinct from direct perception: “what I do is I imagine it in my head, like I can see it but I’m not inside the story or anything” (P20, m11). While children seem to have a clear grasp of the varied feelings and embodied sensations that stories potentially afford, they are emphatic about never having such experiences themselves (#2 –3^**^; #4 –2^**^; #12 –2*; #1 –2): “when someone’s got an injury, in his arm for instance, I don’t feel any of that, no” (P3, f12); “when they’re sad, no, that doesn’t make me sad” (P25, m12).

#### Beyond reading

Text-to-life transfer is primarily about having one’s reading habits, subject interests, and by implication one’s self-understanding, catered to and confirmed. When life-to-text transfer comes up at all, it is mostly in the context of figuring out facts, e.g., when a child (P25, m12) dwells on the geographical details of a story that was loosely set in his hometown. Connected to this, children sharing this perspective are the only group by whom encyclopedias are repeatedly mentioned. Invited life-to-text comparisons (#20 –1) are likewise rejected based on outer, literal dissimilarities: “I’ve only lost two dogs so far and a great-grandma and I can’t say it made me think of them in some way” (P25, m12). Children also comment that they rarely think afterward about what stories do to them, a task that they nevertheless consider potentially uncomplicated: “I don’t do this for anyone, telling them how it makes me feel, what I feel, not even for myself. But I don’t think it’s so hard to describe what I feel” (P20, m11).

### Attachment (F2)

#### Summary

Central to absorbed fiction reading under the *Attachment* perspective is one’s intimate empathic relationship with characters. Children become suspended in following characters’ plights, even to the point of having to downregulate the experience which can be strongly embodied. Equal emphasis is on the text and one’s inner processes. There is a preference for story worlds and characters diverging from everyday reality as these support imaginings that children can augment freely in their minds, beyond the explicitly stated and the ordinary.

#### Reading

Children develop strong emotional bonds (statement #11 ranked + 1^**^; statement #4 ranked + 2; statement #12 ranked + 1) with one or more characters in the story, sometimes to the point of having to downregulate the experience: “for instance if I read a book about animals in Africa being killed then I wouldn’t like – I don’t want to feel that from within” (P7, f11); “or sometimes I might skip to the last page to see the main character’s still there, so I know he doesn’t die” (P10, m12). Empathic feelings are common, particularly in connection with physical suffering and dying but also with less extreme misfortunes such as clashes between friends. At the same time, children remain aware that each character with whom “I choose to bond emotionally” (P10, m12) imposes a unique filter onto their individual story experience: “so through this character you also get to know how the other characters feel” (P22, m11); “but if I liked some other character more, then I’d see it through her (eyes) and I’d understand better why she behaves the way she does” (P18, f12). Protagonists are intensely worried about (#15 –3) – and rejected if they happen to have been replaced between two volumes in a series. In suspenseful moments, the tension arising from such close attachment (#13 + 3; #5 + 2) can be distinctly embodied (e.g., making one sweat, bite one’s lip) and some children locate it in their abdomen specifically: “as if my bowels were tensing up” (P15, f10); “it gives me a total stomachache (…) everything’s just boiling inside me” (P22, m11).

At the same time children overwhelmingly report assuming a spectatorial position, one of an invisible tacit observer who is mostly located inside the story world with the characters (#1 –2; #6 + 2*): “say you’re this person who goes scouting with them so you’re there, except you’re all quiet just watching them, so they have no idea you’re there” (P10, m12). Yet visual imaging is a highly dynamic rather than static experience, either because vantage points shift rapidly as story contents “flash in front of my eyes” (P10, m12) or by virtue of “how the characters move” (P15, f10). Children sharing this perspective prefer to visualize story worlds and characters (#9 + 3*; #6 + 2*) creatively: “so I make an image in my mind but some words don’t feel like they really fit in that room, so I replace them with stuff of my own” (P6, f10). External imagery in illustrations or film adaptations is expressly rejected for curbing this autonomy (#8 –2^**^): “when I imagine the dragons in my head what I see is completely different (from the TV cartoon), like I see these huge spikes on their back and there’s like a dip for the saddle” (P12, f10).

Children sharing this perspective pay close attention to what they read (#16 –2*) and define “a good reader” (#17) in terms of feeling and joy rather than technical skill: “a good reader, I guess everyone has to find out for themselves, but I think it’s more about how much you enjoy it and not so much about how many books you’ve read” (P18, f12). As they focus on immediacy and affect, they are not happy working through comprehension difficulties (#21 0*) which redirect their consciousness to the linguistic medium. Though clearly capable of astute reflection, these children observe an uneasiness about putting their feelings into words and declare that “I wouldn’t know how to describe what it makes me feel” (P1, f11) (#14 0*). This was also reflected in their open pondering of the Q statements that may overall have seemed to them inadequate labels for their inner states.

#### Beyond reading

On various levels, children desire otherness and distinctiveness in books relative to their non-reading life as they know it (life-to-text) but seek out stories that stimulate creative transfer text-to-life (see also section “Discussion” below, split interpretation of #20). These desires are well served by genres such as fantasy, stories set in the distant past, even sci-fi: “I like it when it’s in the future, or in the past, probably things from the past is the best, better than the future, and all kinds of magic too” (P24, m11). These non-realistic genres aside, a more general preference is also expressed for characters whose lives differ from “my life which I can live myself and I don’t have to read about it” (P18, f12) and for narrative renditions of exotic experiences: “say (reading about) a beautiful birthday party, that’s something I’d enjoy feeling” (P7, f11). Stories of such qualities allow one to dream up text-to-life potentialities after reading, in ways that may disregard conventional realism boundaries: “I want to learn how to fight with a sword and stuff like that (…) to fly riding dragons, that must be so cool though there’s downsides to it too” (P12, f10).

### Mental shift (F3)

#### Summary

Central to the Mental Shift perspective is the very process of overcoming distraction, penetrating the linguistic medium and shifting one’s mind into absorption. Children actively work toward speed, technical accuracy, and storyline comprehension which they consider crucial to achieving such shifts; these in turn reward them with an altered perception of time following the specific temporality of the plot. Additional gratifications vary as the group diverges in absorption style. It also diverges in overall attitude to fiction reading and story selection strategies; a strong focus on moment-to-moment achievement is combined here with a focus on text characteristics.

#### Reading

Under the *Mental Shift* perspective, children focus on being perseverant in dealing with written text. They laboriously work out connections between story events, an effort they are willing to make in order to be ultimately carried away by the plot: “so I read it again and then I understand it and then it’s nice again because it all fits in the story and when I like the story overall then all the different bits are good too” (P9, m10). Their notion of “a good reader” (#17) is overwhelmingly linked to speed and accuracy, which they frequently quantify: “say someone who makes zero to three mistakes in a chapter and who reads quite quickly” (P26, m9). Imagining (statement #9 ranked + 1^**^; statement #8 ranked + 1^**^), too, is understood in terms of mental work toward better comprehension (#21 –2) rather than immediate sensory imaging (#6 –1^**^): “when I’m imagining it I’m unpicking the story I’ve just read” (P11, f11); “I’m like teasing apart the different little bits in my head so I can understand it better” (P14, m10). “In (my) head” (P9, m10) or “in the brain” (P8, f9) is also where these children locate reading on the map of their body. Although they deny spending time thinking of particular expressions (#18 –2^**^), they are the group who most frequently offer verbatim quotes from stories and refer to what a formulation “actually says” (P16, f11) as the basis for what they take from it.

Children sharing this perspective are particularly sensitive to moments of losing track, which they consider all too frequent (#3 + 3^**^ understood as low ‘attention,’ see section “Discussion”; #16 + 2^**^): “there must have been like I dunno five or six minutes when I didn’t know what I was reading, my eyes were just moving down the page” (P9, m10); “like when it says ‘Adélka opened the door,’ then I’m just reading on, and by the time I get to ‘the door,’ I don’t remember ‘Adélka opened”’ (P17, f10). Rather than yielding to distraction from external stimuli (#3 + 3^**^ understood as high ‘attention/transportation,’ see section “Discussion”; #19 –3^**^), their mind frequently wanders off the text to other thoughts: “there’s something I’m thinking about but I’m reading at the same time” (P16, f11). Yet they are assiduous in rereading and other strategies to compensate for this: “I go back. A lot. Even four pages sometimes” (P14, m10). Importantly, as their consciousness bounces back and forth within and beyond the text like this, they do not seem to come out of the attentive “bubble” of reading, ultimately losing themselves in the activity for hours (#5 + 3^**^) and enjoying its distinct temporality: “I can completely forget about time and go on reading and I just can’t stop” (P27, m9); “once I read to quarter to eleven because I was so much into the story I forgot to check the clock” (P2, m10).

Though all attest to an altered sense of time, the children diverge as to what else is happening once the shift into a state of absorption has been accomplished. Some sympathize with characters via perspective-taking, some become attached to them empathically, others find it difficult to understand how the characters feel unless it is clearly and simply described in the text. Similar diversity applies to sensory vantage points or life-to-text and text-to-life transfers, and, importantly, to which fiction genres are preferred and how stories are selected. There is a general inclination toward suspenseful page-turners (#13 + 2) which guarantee a speedier flow of time: “The plot must be (…) really exciting and then I just read on and on and on and suddenly it’s nine o’clock instead of eight” (P14, m10). Apart from this, these children seem to ground their selections in whatever makes the shift into absorption most likely to happen for them individually. Some read stories on favorite subjects, some follow selected authors and some may even find it hard at times to leave the confines of a particular series: “when it’s by this author then it’s really nice and easy for me to read” (P8, f9), “I read quite a lot but right now I’ve stopped because I’ve read this book and I can’t find the next one in the series” (P17, f10).

#### Beyond reading

Once absorption is achieved, it consistently blocks out the immediate situation (#19 –3^**^) and reading becomes distinct from life beyond, overriding one’s chores, appointments, and physical needs (#5 + 3^**^): “when I’m reading (…) and granny says I should go pick up lunch then I go back to reading (…) and I forget all about picking up lunch and I just sit there and read” (P14, m10). One child (P8, f9) spontaneously demonstrated how reluctantly she closes her book when life creeps back in and lessons resume after recess in school. Another child says about becoming absorbed that “it’s what I do it for” (P16, f11). The only salient point of contact between reading and life beyond may lie in mentions of other ways of engaging with stories, mostly in films and audiobooks. These are more frequent here than in the remaining groups and in line with the children’s focus on plot and whole-story comprehension over other, more medium-specific experience facets (e.g., sensory imaging): “I listen to audiobooks instead, so I do five chapters in audio and then maybe read two or three” (P9, m10).

## Discussion

Employing a child-centered approach, our Q methodology study identified four distinct perspectives on the lived experience of becoming “lost in a book” among autonomous readers aged 9–12 years. The study addressed multiple research gaps by systematically exploring children’s inner reading experiences which are generally understudied (Wilhelm, 2016) and by contributing to the nascent debate on plurality in readers’ absorption (Kuiken et al., 2022). Incorporating quantitative and qualitative data collection techniques and methods of analysis, we were able to systematically and rigorously study reading experience as shared within each emergent perspective and as varied across the four perspectives (McKeown and Thomas, 2013). Children of both genders, mixed family and school backgrounds, and geographical locations were represented in all four perspectives, and age spans likewise remained wide (F1a: 9–12; F1b: 9–12; F2: 10–12; F3: 9–11). The Q research allowed us to hear a range of voices (Hughes, 2016) and to closely interpret the distinct perspectives.

In this process, the set of cards with authentic experience statements, which the children were invited to sort in a systematic order and then were interviewed about, played a central role. Responding to these stimuli, the children were able to share their perspectives on varied facets of absorbed reading (Kuijpers et al., 2021), describe the workings of reading on a moment-to-moment basis, and reveal how reading relates to their sense of self within and beyond the reading situation. This was naturally integrated with accounts of more specific reading ‘pleasures’ (Wilhelm, 2016), reading motivations (McGeown et al., 2020), overt social interactions (Cremin et al., 2014), and much more. All these accounts were grounded in the structured Q sorts, serving as each child’s frame of reference for understanding the world and their own experience within it (Stephenson, 1953).

For us to understand these internal frames of reference, the children had to be actively invited to interpret the meanings of the individual statements. Two statements were particularly striking as to their interpretation and deserve additional commentary as they demonstrate the unique research affordances of Q methodology. Statement #20 “I want it to resemble my life” was explicitly commented on by seventeen participants. Surprisingly, for ten of these participants, the interpretation ran counter to the statement’s conventional meaning: participants commented on the possibility of living through adventures encountered in books (text-to-life transfer) rather than on recognizing one’s own life in books (life-to-text transfer). Statistically, the statement was not distinguishing or extremely ranked for any factor. However, it was still instrumental to our understanding of the *Attachment* perspective. Here, those who followed the conventional meaning (life-to-text; *n* = 3) evaluated the experience in consistently negative terms while the opposite was true for those assuming the unconventional meaning (text-to-life; *n* = 3). Combined, these contradicting interpretations and inversely contradicting attitudes showed that the group was in fact united in its dislike for all things mundane.

The second notable statement, #3 “I forget where I am when I read,” likewise prompted contradictory interpretations. Of the eighteen participants who commented on this statement, one half (*n* = 9) understood it in terms of *high* ‘attention’ and ‘transportation’ into a world distinct from one’s physical surroundings. This was also the envisioned meaning of the statement (see Table 1), corresponding to what had been expressed in the preliminary research (Kuzmičová et al., 2022). The other relevant participants (*n* = 9) interpreted the statement in terms of *low* ‘attention,’ talking about losing track of the text instead. Unlike #20, this statement did receive extreme composite rankings, in three factors (ranked –3 in F1b; ranked –3 in F2; ranked + 3 in F3). In *Mental Shift*, it was also a statistically distinguishing statement and was discussed in-depth with all but one of nine members. Importantly, the interpretive chasm did not run between the three factors but cut evenly across them. Without the post-sorting comments on this statement, its dual nature, and concomitant experience complexity especially in *Mental Shift*, would not have become evident.

The divergent statement interpretations illustrate the main contribution as well as the limitations of our Q study and its inductive quantitative-qualitative principles. On the one hand, the study allowed us to clearly demonstrate that there is not one universal way of being absorbed in reading and that research inviting children to openly share their distinct perspectives is needed. On the other hand, the invitation for participants to supply their own meaning, or “psychological significance,” to the statements complicates the analytical and interpretive processes (Watts and Stenner, 2012, p. 70). It is the researcher who is responsible for recognizing any contradictory interpretations and arriving at a holistic understanding of the data, while at the same time ensuring that the participants rather than the researcher remain central to the study (*ibid*.). Providing a concise overview of the research and an easily applicable blueprint for future work then proves difficult because in Q methodology, data is not simply collected but rather emerges in the participant’s reciprocal interactions with the stimuli (interpretation, comparison, sorting) as well as the researcher (explaining extreme rankings, discussing one’s interpretations).

The different dimensions of absorption as shown in Table 1 represent one of many possible ways of reducing our findings for the sake of discussion. Each composite Q sort accentuates slightly different dimensions. Looking at highly ranked (+3, +2) positive (i.e., non-reverse) items through the lens of the Story World Absorption Scale (SWAS; Kuijpers et al., 2014), for instance, the *Mental Shift* perspective (Table 5) gravitates towards the scale’s ‘attention’ and ‘transportation’ dimensions. For the *Confirmation* perspective (Table 3), the only highly ranked positive item linked to absorption concerns ‘mental imagery;’ all other positive items in the plus area fall outside absorption as defined by the SWAS or other tools. For the *Growth* (Table 2) and *Attachment* (Table 4) perspectives in turn, the plus area of the grid displays more complex combinations of ‘mental imagery,’ ‘emotional engagement’ and ‘transportation’ items. These combinations are not sufficiently described using SWAS categories alone and may require the more fine-grained concepts offered by the Absorption-like States Questionnaire (ASQ; Kuiken et al., 2022). However, as shown under “Results”, both perspectives cut across the two alternative absorption routes distinguished by the ASQ. In *Growth*, the expressive enactment route clearly dominates when it comes to characters’ embodied sensations; characters’ emotions on the other hand seem to call forth integrative comprehension. The inverse applies to the *Attachment* perspective.

Importantly, no two perspectives differed just along the experience dimensions captured in measures of absorption (Kuijpers et al., 2014; Kuiken et al., 2022). Rather, much more deep-going differences emerged that spoke to divergent roles of reading in one’s life and concomitant divergent workings of one’s consciousness while reading and reflecting on reading. This is particularly true of the first three perspectives, *Growth, Confirmation*, and *Attachment*. These perspectives stand in opposition to each other but together also differ in kind from the last one, *Mental Shift*, insofar as they point beyond the realm of reading: to expanding (*Growth*) or not (*Confirmation*) of one’s boundaries as a person and to empathizing with (fictional) others (*Attachment*). Meanwhile, *Mental Shift* suggests an angle on absorbed reading that has limited bearing beyond mastering reading as such. We propose that the children in this group stand yet to transition into the remaining perspectives. The *Mental Shift* perspective is thus a reminder that the emergent perspectives should not be understood as fixed identity labels but as perspectives which may change, even repeatedly and in more than one direction, as fiction reading styles indeed continue to evolve into adulthood (Charlton et al., 2004).

Many of the core experiences defining the *Growth, Confirmation*, and *Attachment* perspectives have been identified in previous studies: *Growth* largely conforms to the complex of “transformative dialogic” reading strategies that have been described in education research (Schrijvers et al., 2019); *Confirmation* as a group are directly concerned by calls for enabling personalized subject-driven reading for pleasure in schools (Cremin et al., 2014); the reading style of *Attachment* links to a rich tradition of research across disciplines into young readers’ empathy (Kucirkova, 2019) and identification (Andringa, 2004) and other character-driven modes of story engagement (Calvert and Richards, 2014; Rain et al., 2017). In all these cases, our Q methodology study enabled fleshing out a child-centered and more rounded view of familiar facts of people’s lives with books and other media. Meanwhile, *Mental Shift* as a distinct perspective within young fiction readers’ “universe of subjectivity” (Brown et al., 2015) is an uncharted territory and requires further systematic research unpacking its experiential components, not least because it marks the potentially most vulnerable and volatile reader group from an educational point of view.

Children within the *Mental Shift* perspective were also the most consistent in their interpretation of statement #17 “I am a good reader.” For them, the item exclusively suggested technical efficacy, in line with how the very same statement is classified in the Motivation for Reading Questionnaire (Wigfield and Guthrie, 1997) - and in line with how “good reading” is often reductively understood in schools (Cremin et al., 2014; Mackey, 2022). By contrast, children in all the other perspectives tended at least partly to either disregard a technicist definition or complement it with additional qualities: one’s private pleasures, reflective depth, openness to different fiction genres, and so forth. Moreover, for the *Mental Shift* group, striving to shift gears onto the level of “a good reader” seemed to also affect their self-perception in the longer term.

As a key invitation for future research, it is therefore desirable that new child-centered studies focus on the longer-term shifts, soliciting children’s accounts of having recently become a “better” reader and how this registers in their moment-to-moment reading experience. Until such work is accomplished, our key practical conclusion is that rather than being led to focus on efficacy, children should be taught to reflect on the fuller range of routes to absorbed reading, and on the fact that these vary across individuals.

## Data availability statement

The raw data supporting the conclusions of this article will be made available by the authors, without undue reservation.

## Ethics statement

The studies involving human participants were reviewed and approved by the Research Ethics Committee of the Faculty of Arts, Charles University. Written informed consent to participate in this study was provided by the participants’ legal guardian/next of kin. Written informed consent was obtained from the individual(s) for the publication of any potentially identifiable images or data included in this article.

## Author contributions

AK designed the overall structure and drafted and finalized the full manuscript. MS co-drafted key sections (“Materials and methods,” “Results,” and “Discussion”) and made revisions to the remaining manuscript. MN co-drafted key sections (“Materials and methods”) and made revisions to selected sections (“Results” and “Discussion”). All authors took part in major conceptual decisions regarding the manuscript.
